# A new genome sequence resource for five invasive fruit flies of agricultural concern: *Ceratitis capitata*, *C. quilicii*, *C. rosa*, *Zeugodacus cucurbitae *and *Bactrocera zonata *(Diptera, Tephritidae)

**DOI:** 10.12688/f1000research.157946.1

**Published:** 2024-12-06

**Authors:** Pablo Deschepper, Sam Vanbergen, Lore Esselens, John S. Terblanche, Minette Karsten, Maxi Snyman, Domingos Cugala, Laura Canhanga, Luis Bota, Maulid Mwatawala, Majubwa Ramadhani, Abdul Kudra, Jenipher Tairo, Jacqueline Bakengesa, Pia Addison, Aruna Manrakhan, Corentin Gledel, Hélène Delatte, Marc De Meyer, Massimiliano Virgilio

**Affiliations:** 1 Biology department, invertebrates section, Royal Museum for Central Africa, Tervuren, Belgium; 2 Stellenbosch University Department of Conservation Ecology and Entomology, maiteland, South Africa; 3 University of Eduardo Mondlane College of Agriculture and Forestry, Maputo, Maputo City, Mozambique; 4 Centre of Excellence in Agri-Food Systems and Nutrition, University of Eduardo Mondlane, Maputo, Mozambique; 5 Provincial Directorate of Agriculture and Food Security, National Fruit Fly Laboratory, Chimoio, Manica, Mozambique; 6 Department of Crop Science and Horticulture, Sokoine University of Agriculture, Morogoro, Morogoro Region, Tanzania; 7 Department of Biology, The University of Dodoma, Dodoma, Dodoma Region, Tanzania; 8 Citrus Research International Pty Ltd, Nelspruit, Mpumalanga, South Africa; 9 CIRAD, UMR PVBMT, Saint-Pierre, La Réunion, 97410, France

**Keywords:** genome assembly, invasive species, fruit fly, tephritidae, pest

## Abstract

Here, we present novel high quality genome assemblies for five invasive tephritid species of agricultural concern: *Ceratitis capitata*, *C. quilicii*, *C. rosa*, *Zeugodacus cucurbitae* and *Bactrocera zonata* (read depths between 65 and 78x). Three assemblies ( *C. capitata*, *C. quilicii* and *Z. cucurbitae*) were scaffolded with chromosome conformation data and annotated using RNAseq reads. For some species this is the first reference genome available ( *B. zonata*, *C. quilicii* and *C. rosa*), for others we have published improved annotated genomes ( *C. capitata* and *Z. cucurbitae*). Together, the new references provide an important resource to advance research on genetic techniques for population control, develop rapid species identification methods, and explore eco-evolutionary studies.

## Introduction

A significant number of phytophagous insects within the dipteran family of the Tephritidae (the “true” fruit flies) are considered as serious pests for fruits and vegetables worldwide ( White & Elson-Harris 1992). Globalization has led to a surge in intercontinental trade and movement, and has increased the number of incursions of harmful non-native fruit fly species ( Bragard *et al.* 2020). Many countries have put costly and elaborate phytosanitary measures in place to prevent entry and establishment of harmful fruit fly species ( Bragard *et al.* 2020; Papadopoulos *et al.* 2023a, 2023b). Making resources available that could provide researchers with a better tool for studying fruit fly pests is becoming increasingly important. Agricultural areas with a suitable climate for fruit fly pests are rapidly increasing around the globe ( Sultana *et al*. 2020), changing patterns of distribution of fruit fly pests ( Ni *et al*. 2011). This leads to more fruit fly incursions and first detections of new fruit fly species in several countries in recent years, e.g. *B. dorsalis* in France, Italy and Belgium; *B. zonata* in France (EPPO alert list, https://www.eppo.int/ACTIVITIES/plant_quarantine/alert_list).

**Figure 1.**
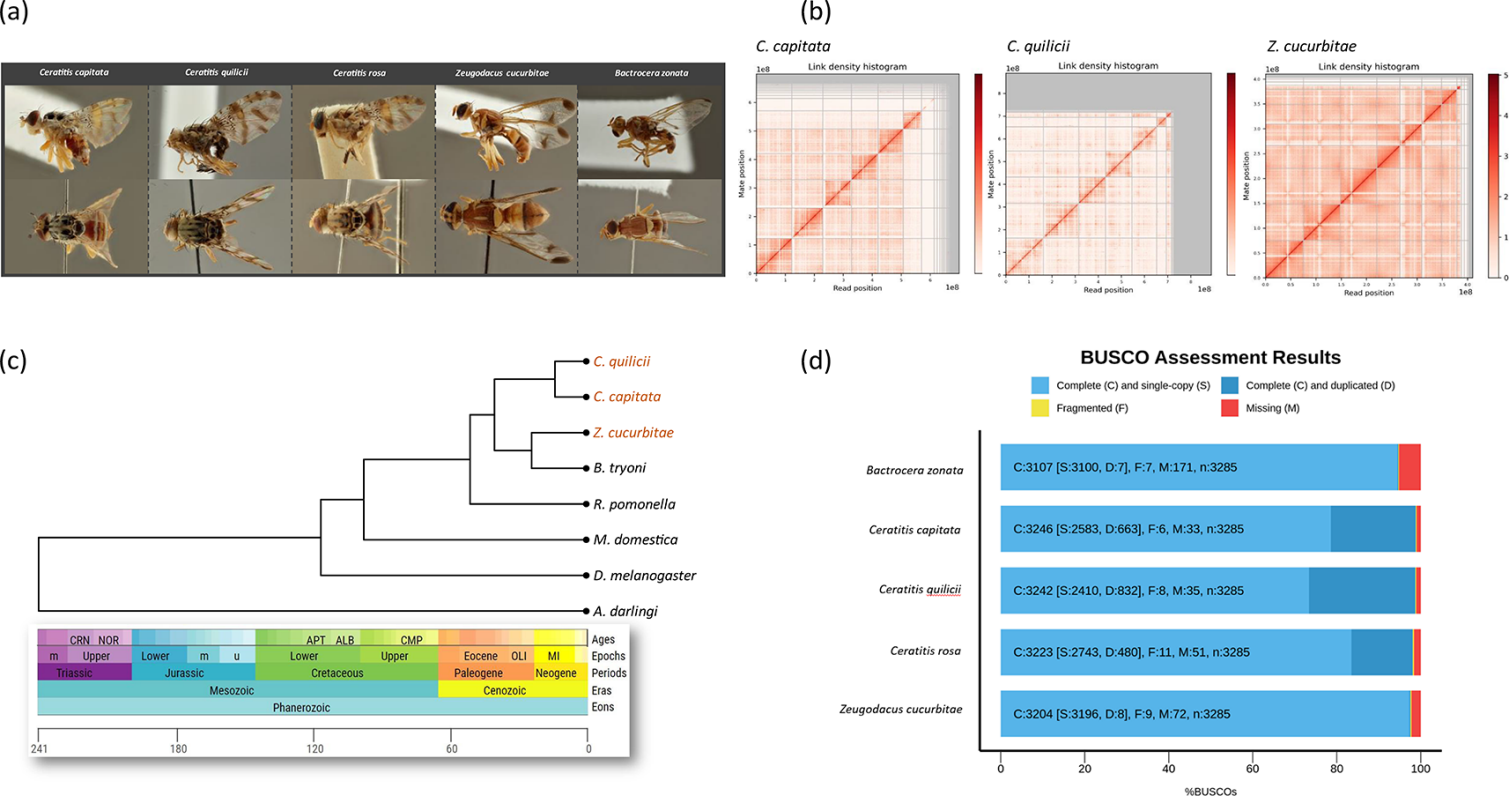
Linkage and BUSCO completeness across the genomes presented here and phylogenetic analysis. (a) Photographs of the five fruit fly pest species from a dorsal and lateral view © RMCA (Royal Museum for Central Africa. (b) Hi-C (Dovetail™ Omni-C™) contact map for three tephritid species showing which reads are in close proximity of each other, revealing the linear representation of the scaffolds/chromosomes within the genome. (c) Phylogenetic tree of the three tephritid fruit flies with annotation and five other diptera species. (d) BUSCO completeness results for each of the assembled tephritid genomes.

Here, we present high quality reference genome assemblies for five tephritids ( *Ceratitis capitata* (Wiedemann), *C. quilicii* (De Meyer, Mwatawala & Virgilio), *C. rosa* (Karsch), *Zeugodacus cucurbitae* (Coquillett), *Bactrocera zonata*) of agricultural importance ( Figure 1a). For three ( *C. quilicii, C. rosa*, *B. zonata*) of the five species, a genome assembly is completely lacking in public databases and could thus provide a major step forward in accumulating knowledge on those species. Genome assemblies are a valuable resource for both fundamental and applied research and can facilitate the development of new and sustainable pest management methods. The highly contiguous and complete genomes presented here will increase the chances of researchers to find specific genes of interest and investigate changes in genomic architecture. The new assemblies will enable researchers to tackle questions regarding climate adaptation, host and range expansion and niche shifts ( Papanicolaou *et al.* 2016).

## Results and discussion

PacBio CSS reads covered the genome between 65 and 78 times assuming a genome size of 0.5 Gb ( Table 1) for the five fruit fly species shown in Figure 1a. A BUSCO search for genome completeness for all five novel assemblies against the Diptera database delivered a decent genome completeness between 94.6% ( *B. zonata*) and 98.8% ( *C. capitata*) using the duplicate purged PacBio assemblies ( Figure 1d). Total assembly lengths ranged from 410 Mb ( *Z. cucurbitae*) to 889 Mb ( *C. quilicii*) with L50 values ranging from three ( *B. zonata*) to 63 ( *C. quilicii)* ( Table 1). BlobToolKit results for identifying contaminants are shown in Figure. S1-S5 (Refer extended data) accessible at https://zenodo.org/records/14186560). Physical pairing between chromatin regions is shown in Figure 1b for *C. capitata*, *C. quilicii* and *Z. cucurbitae.*

**Table 1.** Comparison of assembly statistics.

	PacBio CSS read data			genome length and contiguity				
Species	Number of Reads	Bp (Gb)	Coverage	Total Length (bp)	N50	L50	N90	L90
*Ceratitis capitata*	2,563,883	38.8	78x	699,814,289	8,193,440	25	817,442	121
*Ceratitis quilicii*	2,498,505	38.4	77x	889,108,370	4,088,374	63	278,990	378
*Ceratitis rosa*	2,451,133	36.2	72x	650,940,389	17,660,867	12	966,435	75
*Zeugodacus cucurbitae*	2,332,131	32.4	65x	410,169,932	14,989,679	8	4,412,789	28
*Bactrocera zonata*	2,522,876	32.4	65x	524,894,629	99,542,525	3	22,789,729	6

The annotated genomes comprise 32,449; 38,590 and 31,422 genes in total for *C. capitata*, *C. quilicii* and *Z. cucurbitae* respectively with a total coding region length (bp) of 39,037,294; 46,768,995 and 41,286,253. The average gene length (bp) is 1,203.04; 1,211.95 and 1,313.93 for *C. capitata*, *C. quilicii* and *Z. cucurbitae* respectively. The most recent *C. capitata* assembly available on NCBI (GCA_905071925.1, published in November 2020) contains 14,054 genes and thus, this novel assembly improves the degree of annotation of the *C. capitata* genome significantly. The same can be observed in *Z. cucurbitae*, where the most recent NCBI reference assembly (GCF_028554725.1) only comprises 17,225 genes. In *Ceratitis* sp. however, a substantial proportion of BUSCO’s are duplicated, which suggest the presence of redundant sequences resulting from partial misassemblies. Our recommendation is therefore to be cautious when comparing *Ceratitis* sp. assemblies with other assemblies.

A total of 19,480 gene orthogroups could be found using OrthoFinder and a total of 32,051; 37,950 and 31,009 genes could be attributed to an orthogroup for *C. capitata*, *C. quilicii* and *Z. cucurbitae* respectively. Using these orthogroups as evidence we estimated that the Tephritidae-Drosophilidae split took place around 120 MYA ( Figure 1c), which is in line with the estimations of Russo *et al*. (2013) who constructed a drosophilid time tree with two tephritid species as outgroup ( *C. capitata* and *B. oleae*) and estimated the split at around 110 MYA.

We believe that our contribution will substantially impact tephritid genome research and provides new opportunities for comparative genomics with a focus on characterizing genes related to invasiveness.

## Methods

### *De novo* genome assembly

An inbred lab colony of each of the following tephritid species was established in an artificial setting and larvae were collected for subsequent sequencing: *Ceratitis capitata*, *C. quilicii*, *C. rosa*, *Zeugodacus cucurbitae* and *Bactrocera zonata.* Inbred specimens of *C. quilicii*, *C. capitata* and *C. rosa* were produced at Citrus Research International in Mbombela and were originally sourced from wild flies collected in Ermelo (-26.516021, 29.996168), Burgershall (-25.112083, 31.087778) and Mbombela (-25.452258, 30.970778), Mpumalanga Province, South Africa respectively in 2020 ( *C. rosa*) and 2021 ( *C. capitata* and *C. quilicii*). Species identity was confirmed by Marc De Meyer ( *C. quilicii*) and Aruna Manrakhan ( *C. capitata* and *C. rosa*). Inbred lines for *Z. cucurbitae* and *B. zonata* were already present at the facilities of CIRAD, Réunion for more than 150 generations and could thus be used for our purposes. Pupae of all species supplied for sequencing originate from a parent x F1 backcross to increase homozygosity. The sequencing and assembly process can be described by three consecutive steps: generation of PacBio CCS reads and primary assembly with Hifiasm, generation of Hi-C (specifically, Dovetail™ Omni-C™ reads) coupled with secondary assembly using HiRise and lastly, generation of an RNAseq library for ab initio genome annotation. Only the assemblies of *C. capitata*, *C. quilicii* and *Z. cucurbitae* comprised the HiRise scaffolding and annotation steps.

*De novo PacBio assembly and filtering*

A *de novo* assembly was constructed using ±38.8 Gb of PacBio CCS reads resulting in a coverage of around 70x of the tephritid genome ( Table 1). The obtained PacBio reads were used as input to Hifiasm v0.15.4-r347 ( Cheng *et al.* 2021) with default parameters. Blast results of the Hifiasm output assembly against the nucleotide BLAST database ( https://blast.ncbi.nlm.nih.gov/) were used as input for blobtools v1.1.1 ( Laetsch and Blaxter 2017) and scaffolds identified as possible contamination were removed from the assembly. Finally, purge_dups3 v1.2.5 ( Guan *et al.* 2020) was used to purge haplotigs and contig overlaps. The final assembly was checked for its completeness using BUSCO using the diptera_odb10 dataset ( Manni *et al.* 2021).

*Chromosome conformation capture and HiRise scaffolding*

To construct a Dovetail™ Omni-C™ library, chromatin was fixed in place with formaldehyde in the nucleus and then extracted. Fixed chromatin was digested with DNAse I, chromatin ends were repaired and ligated to a biotinylated bridge adapter followed by proximity ligation of adapter containing ends. After proximity ligation, crosslinks were reversed and the DNA purified. Purified DNA was treated to remove biotin that was not internal to ligated fragments. Sequencing libraries were generated using NEBNext Ultra enzymes and Illumina-compatible adapters. Biotin-containing fragments were isolated using streptavidin beads before PCR enrichment of each library. The library was sequenced on an Illumina HiSeqX platform to produce approximately 30x sequence coverage.

The input de novo assembly and Dovetail™ Omni-C™ library reads (MQ > 50) were used as input data for HiRise, a software pipeline designed specifically for using proximity ligation data to scaffold genome assemblies ( Putnam *et al.* 2016). Dovetail™ Omni-C™ library sequences were aligned to the draft input assembly using bwa ( https://github.com/lh3/bwa). The separations of Dovetail™ Omni-C™ read pairs mapped within draft scaffolds were analyzed by HiRise to produce a likelihood model for genomic distance between read pairs, and the model was used to identify and break putative misjoins, to score prospective joins, and make joins above a threshold.

### *Ab initio* genome annotation

Firstly, repeat families in the three tephritid genome assemblies ( *C. capitata*, *C. quilicii* and *Z. cucurbitae*) were identified *de novo* and classified using the software package RepeatModeler2 ( Flynn *et al.* 2020, the original version of RepeatModeler is free and available at https://github.com/Dfam-consortium/RepeatModeler/blob/master/RepeatModeler). The custom repeat library obtained from RepeatModeler2 was used to discover, identify and mask the repeats in the assembly using RepeatMasker (Version 4.1.0, available at https://github.com/rmhubley/RepeatMasker). Secondly, coding sequences from *Bactrocera dorsalis*, *Ceratitis capitata* and *Drosophila melanogaster* available on GenBank were used to train the *ab initio* model in AUGUSTUS (version 2.5.5) by performing six rounds of optimization. Likewise, the same coding sequences were used to train an independent *ab initio* gene model using SNAP ( Korf 2004). Furthermore, RNAseq reads were mapped onto the genome using the STAR aligner software ( Dobin *et al.* 2013). MAKER ( Campbell *et al.* 2014), SNAP and AUGUSTUS (with intron-exon boundary hints provided from RNAseq) were then used to predict genes in the repeat-masked reference genome. To help guide the prediction process, SwissProt peptide sequences from the UniProt database ( https://www.uniprot.org/) were downloaded and used in conjunction with the protein sequences from the aforementioned species to generate peptide evidence in the Maker pipeline ( Campbell *et al.* 2014). Only genes that were predicted by both SNAP and AUGUSTUS were retained in the final gene sets. To help assess the quality of the gene prediction, AED scores were generated for each of the predicted genes as part of the MAKER pipeline. Genes were further characterised for their putative function by performing a BLAST ( Ye *et al.* 2006) search of the peptide sequences against the UniProt database. tRNA were predicted using the software tRNAscan-SE ( Lowe & Chan 2016, available at: https://lowelab.ucsc.edu/tRNAscan-SE/).

### Phylogenetic tree reconstruction

We inferred orthogroups using OrthoFinder v2.5.5. ( Emms & Kelly 2019) for the three fruit fly species with an annotated genome assembly in this study ( *C. capitata*, *C. quilicii* and *Z. cucurbitae*). In addition, we downloaded protein sequence data for *Drosophila melanogaster* Meigen (GCA_000001215.4), *Anopheles darlingi* Root (GCA_000211455.3), *Musca domestica* Linnaeus (GCF_030504385.1), *Rhagoletis pomonella* (Walsh) (GCF_013731165.1) and *Bactrocera tryoni* (Froggatt) (GCF_016617805.1). Sequences were aligned using Diamond and gene trees were inferred using fasttree. The STAG algorithm combined with the STRIDE rooting methods, implemented in OrthoFinder, was then used to infer a species tree with realistic branch lengths from the full set of gene trees ( Emms & Kelly 2017). A time-calibrated tree was constructed by transforming the species tree rendered by Orthofinder into a ultrametric tree and calibrating it based on the split between *A. darlingi* and the rest of the taxa (240.8 MYA) as inferred from TIMETREE5 ( timetree.org).

## Author contributions

PD, SV, LE, MDM, MV (RMCA, BE) – Conceptualization, funding acquisition, original draft preparation and data submission.

PA, JT, MK (SU, ZA) – Conceptualization, development of the inbred lines, provision of field samples, review and editing.

AM (CRI, ZA) - Conceptualization, development of the inbred lines, provision of field samples, review and editing.

DC, LC (EMU, MZ), LB (National FF lab, MZ) - Conceptualization, provision of field samples, review and editing.

MM, RM, AK, JT (SUA, TZ), JB (UDOM, TZ) - Conceptualization, review and editing.

HD (CIRAD – La Réunion, FR) - Conceptualization, funding acquisition, development of the inbred lines, provision of field samples, review and editing.

## Data Availability

All five genome assemblies have been deposited on the NCBI data repository. National Centre for Biotechnology Information. BioProject: Five new genome assemblies of Tephritid pest species. Accession number: PRJDB18489; https://www.ncbi.nlm.nih.gov/bioproject/PRJDB18489/. GenBank assemblies for the five tephritid species can be consulted using following identifiers: National Centre for Biotechnology Information. GCA_043005645.1: *Bactrocera zonata*; https://www.ncbi.nlm.nih.gov/datasets/genome/GCA_043005645.1/. National Centre for Biotechnology Information. GCA_043005455.1: *Ceratitis capitata*; https://www.ncbi.nlm.nih.gov/datasets/genome/GCA_043005455.1/. National Centre for Biotechnology Information. GCA_043005495.1: *Ceratitis quilicii*; https://www.ncbi.nlm.nih.gov/datasets/genome/GCA_043005495.1/. National Centre for Biotechnology Information. GCA_043005725.1: *Ceratitis rosa*; https://www.ncbi.nlm.nih.gov/datasets/genome/GCA_043005725.1/. National Centre for Biotechnology Information. GCA_043005565.1: *Zeugodacus cucurbitae*; https://www.ncbi.nlm.nih.gov/datasets/genome/GCA_043005565.1/ Annotation files for *C. capitata*, *C. quilicii* and *Z. cucurbitae* are stored at zenodo. https://zenodo.org/records/13928607, Genome sequence and .gff annotation of three pest fruit flies (Tephritidae). zenodo. Genome sequence and .gff annotation of three pest fruit flies (Tephritidae), DOI: https://doi.org/10.5281/zenodo.13928607 ( Royal Museum for Central Africa 2024). The project contains the following underlying data:
•

Zcucurbitae_DDBJ_100Ngaps.gff
•

Zcucurbitae_DDBJ_100Ngaps.fa
•

Cquilicii_DDBJ_100Ngaps.gff
•

Cquilicii_DDBJ_100Ngaps.fa
•

Ccapitata_DDBJ_100Ngaps.gff
•

Ccapitata_DDBJ_100Ngaps.fa Data are available under the terms of the Creative Commons Attribution 4.0 International license (CC-BY 4.0). Zenodo: A new genome sequence resource for five invasive fruit flies of agricultural concern: Ceratitis capitata, C. quilicii, C. rosa, Zeugodacus cucurbitae and Bactrocera zonata (Diptera, Tephritidae), DOI: https://doi.org/10.5281/zenodo.14186560 ( Deschepper 2024). The project contains the following extended data:
• FigS1.png • FigS2.png • FigS3.png • FigS4.png • FigS5.png Data are available under the terms of the Creative Commons Attribution 4.0 International license (CC-BY 4.0).

## References

[ref1] Bragard C EFSA Panel on Plant Health (PLH) : Pest categorisation of non-EU Tephritidae. *EFSA J.* 2020. 10.2903/j.efsa.2020.5931

[ref2] Campbell MS Holt C Moore B : Genome Annotation and Curation Using MAKER and MAKER-P. *Curr. Protoc. Bioinformatics.* 2014;48:4.11.1–4.11.39. 10.1002/0471250953.bi0411s48 25501943 PMC4286374

[ref3] Cheng H Concepcion GT Feng X : Haplotype-resolved de novo assembly using phased assembly graphs with hifiasm. *Nat. Methods.* 2021;18:170–175. 10.1038/s41592-020-01056-5 33526886 PMC7961889

[ref22] Deschepper P : A new genome sequence resource for five invasive fruit flies of agricultural concern: Ceratitis capitata, C. quilicii, C. rosa, Zeugodacus cucurbitae, and Bactrocera zonata (Diptera, Tephritidae).[Dataset]. *Zenodo.* 2024. 10.5281/zenodo.14186560 PMC11871432 40028447

[ref4] Dobin A Davis CA Schlesinger F : STAR: Ultrafast universal RNA-seq aligner. *Bioinformatics.* 2013;29:15–21. 10.1093/bioinformatics/bts635 23104886 PMC3530905

[ref5] Emms DM Kelly S : STRIDE: Species Tree Root Inference from Gene Duplication Events. *Mol. Biol. Evol.* 2017;34:3267–3278. 10.1093/molbev/msx259 29029342 PMC5850722

[ref6] Emms DM Kelly S : OrthoFinder: phylogenetic orthology inference for comparative genomics. *Genome Biol.* 2019;20:238. 10.1186/s13059-019-1832-y 31727128 PMC6857279

[ref8] Flynn JM Hubley R Goubert C : RepeatModeler2 for automated genomic discovery of transposable element families. *Proc. Natl. Acad. Sci.* 2020;117:9451–9457. 10.1073/pnas.1921046117 32300014 PMC7196820

[ref9] Korf I : Gene finding in novel genomes. *BMC Bioinformatics.* 2004;5. 10.1186/1471-2105-5-59 15144565 PMC421630

[ref10] Laetsch DR Blaxter ML : BlobTools: Interrogation of genome assemblies [version 1; peer review: 2 approved with reservations]. *F1000Res.* 2017;6. 10.12688/f1000research.12232.1

[ref11] Lowe TM Chan PP : tRNAscan-SE On-line: Integrating search and context for analysis of transfer RNA genes. *Nucleic Acids Res.* 2016;44:W54–W57. 10.1093/nar/gkw413 27174935 PMC4987944

[ref12] Guan D McCarthy SA Wood J : Identifying and removing haplotypic duplication in primary genome assemblies. *Bioinformatics.* 2020;36:2896–2898. 10.1093/bioinformatics/btaa025 31971576 PMC7203741

[ref13] Manni M Berkeley MR Seppey M : BUSCO: Assessing genomic data quality and beyond. *Current Protocols.* 2021;1:e323. 10.1002/cpz1.323 34936221

[ref14] Ni WL Li ZH Chen HJ : Including climate change in pest risk assessment: the peach fruit fly, *Bactrocera zonata* (Diptera: Tephritidae). *Bull. Entomol. Res.* 2011;102:173–183. 10.1017/S0007485311000538/ 22008216

[ref15] Papadopoulos NT Camilleri M Graziosi I : Surveillance of non-EU Tephritidae in the EU: a guide for using EFSA pest survey cards. *EFSA Supporting Publications.* 2023a. 10.2903/sp.efsa.2023.EN-7773

[ref16] Papadopoulos NT De Meyer M Terblanche JS : Fruit flies: challenges and opportunities to stem the tide of global invasions. *Annu. Rev. Entomol.* 2023b;69:355–373. 10.1146/annurev-ento-022723-103200 37758223

[ref17] Papanicolaou A : The whole genome sequence of the Mediterranean fruit fly, *Ceratitis capitata* (Wiedemann), reveals insights into the biology and adaptive evolution of a highly invasive pest species. *Genome Biol.* 2016;17:192. 10.1186/s13059-016-1049-2 27659211 PMC5034548

[ref18] Putnam NH O'Connell BL Stites JC : Chromosome-scale shotgun assembly using an in vitro method for long-range linkage. *Genome Res.* 2016;26:342–350. 10.1101/gr.193474.115 26848124 PMC4772016

[ref24] Royal Museum for Central Africa Vanbergen S Esselens L : Genome sequence and .gff annotation of three pest fruit flies (Tephritidae). *Zenodo.* 2024. 10.5281/zenodo.13928607

[ref23] Russo CAM Mello B Frazão A : Phylogenetic analysis and a time tree for a large drosophilid data set (Diptera: Drosophilidae). *Zool. J. Linn. Soc.* 1 December 2013;169(4):765–775. 10.1111/zoj12062

[ref19] Sultana S Baumgartner JB Dominiak BC : Impacts of climate change on high priority fruit fly species in Australia. *PLoS One.* 2020;15:e0213820. 10.1371/journal.pone.0213820 32053591 PMC7018044

[ref20] White IM Elson-Harris MM : *Fruit flies of economic significance: their identification and bionomics.* Wallingford, UK: CAB International;1992. 9780851987903.

[ref21] Ye J McGinnis S Madden TL : BLAST: Improvements for better sequence analysis. *Nucleic Acids Res.* 2006;34:W6–W9. 10.1093/nar/gkl164 16845079 PMC1538791

