# Supplementary material for: A new genome sequence resource for five invasive fruit flies of agricultural concern: Ceratitis capitata, C. quilicii, C. rosa, Zeugodacus cucurbitae and Bactrocera zonata (Diptera, Tephritidae)
Source: F1000Res. 2024 Dec 6;13:1492. [Version 1] doi: 10.12688/f1000research.157946.1 (PMC11871432; doi:10.12688/f1000research.157946.1)
Supplement: Peer review history

# Reviewer report: Reviewer response for version 1

**DOI:** 10.5256/f1000research.173473.r365152

**Published:** 2025-02-28

**Authors:** Lucio Navarro Escalante

**Affiliations:** 1 The University of Texas at Austin, Austin, USA

Report:

 The manuscript reports the sequencing and assembly for the genomes of five tephritid fly species of agricultural importance (Ceratitis capitata, .C. quilicii, C. rosa, Bactrocera zonata and Zeugodacus cucurbitae). PacBio sequences were used to assembly contig level high quality draft genomes for the five species with completeness ranged between 95%-99%. Three of them (C. capitata, C. quilicii and Z. cucurbitae) were further scaffolded using Hi-C chromosome conformation data. Additionally, for three of these flies (C. quilicii, C. rosa and B. zonata) this data represents the first reported assembled genome. RNAseq data was also produced and used to predict gene contents in scaffolded genomes (C. capitata, C. quilicii and Z. cucurbitae) by performing a combination of ab-initio and evidence-based methods. Finally, a phylogenetic tree was built with the annotated genomes using orthologous protein sequences as evidence.

 The significance for the sequenced species is clearly defined in the manuscript. The described methods offer enough details and are appropriate for the goals, however part of the datasets does not seem to be fully available. Specifically, I have not been able to find the original raw genome sequences (PacBio and Hi-C Illumina libraries), nor the manuscript indicating where this data is stored. Authors should provide SRA accession numbers for this raw data.

 Minor comments:

- Are there any references about the number of chromosomes in these particular fly species? If so, that should be reported and could be used to discuss any correlation with the number of chromatin regions detected in the Hi-C interaction matrix. In fact, the authors do not provide any detail or discussion about these observations in the Hi-C analysis.
- The total percentage of repetitive sequences for each assembled genome should be also reported.
- Authors should simplify users' access to data by providing FASTA files containing the predicted protein and CDS sequences for the annotated genomes in this study.
- Include more details about the methods used for DNA extraction, at least indicate what type of method was used.
- For the ab initio genome annotation methods, specify the meaning of 'AED’.
- In supplementary figures S1-S5, each image should clearly indicate to what fly species they correspond. Additionally, images with better resolution should be provided.
- Check for missing or spare parenthesis across the manuscript.

Are the datasets clearly presented in a usable and accessible format, and the assembly and annotation available in an appropriate subject-specific repository?

Partly

Are sufficient details of the sequencing and extraction, software used, and materials provided to allow replication by others?

Yes

Are the rationale for sequencing the genome and the species significance clearly described?

Yes

Are the protocols appropriate and is the work technically sound?

Yes

Reviewer Expertise:

Insect genomics

I confirm that I have read this submission and believe that I have an appropriate level of expertise to confirm that it is of an acceptable scientific standard.

# Reviewer report: Reviewer response for version 1

**DOI:** 10.5256/f1000research.173473.r365150

**Published:** 2025-02-26

**Authors:** Craig Wilding

**Affiliations:** 1 Liverpool John Moores University, Liverpool, England, UK

Deschepper et al. describe the production of high-quality chromosomal assemblies for each of five invasive fruit flies. This is an excellent body of work comprising not just PacBio sequencing, but Hi-C data.

 The methods used are both clearly described and appropriate, and produced good to excellent (dependent upon species) results in terms of genome contiguity and annotation completeness. There are differences in quality of assemblies, as adjudged by BUSCO data where two species assemblies have substantial apparent duplicated genes (which the authors discuss) but, nevertheless, these are good quality genomes which will be of value to those studying these species from a control perspective, as well as in understanding their relationships.

**Comments:**

- The statement in the results that there is "a decent genome completeness" is vague and non-quantitative. I would avoid vague terms like decent.
- The use of BlobTools is mentioned in the methods but there is then no comment on the output from this in the results and no blobplot provided. What did it show? (Perhaps some of these flies have, for instance, *Wolbachia*?)
- Why are Hi-C maps provided for just 3 of the 5 genomes?
- I am unclear on the point of Fig 1c given its limited number of species. What is the message from this?
- Aside from the differences in gene content for the new versus existing *C. capitata* and *Z. cucurbitae* assemblies, how different are these in other parameters e.g. gene identity of coding genes? Will there be any future attempt to utilise these assemblies in combination to examine variability such as SNPs and CNVs?

Are the datasets clearly presented in a usable and accessible format, and the assembly and annotation available in an appropriate subject-specific repository?

Yes

Are sufficient details of the sequencing and extraction, software used, and materials provided to allow replication by others?

Yes

Are the rationale for sequencing the genome and the species significance clearly described?

Yes

Are the protocols appropriate and is the work technically sound?

Yes

Reviewer Expertise:

Evolutionary genetics

I confirm that I have read this submission and believe that I have an appropriate level of expertise to confirm that it is of an acceptable scientific standard.
